# Mapping the use of research to support strategies tackling maternal and child health inequities: evidence from six countries in Africa and Latin America

**DOI:** 10.1186/s12961-015-0072-1

**Published:** 2016-01-07

**Authors:** Emily Vargas, Victor Becerril-Montekio, Miguel Ángel Gonzalez-Block, Patricia Akweongo, Cynthia N. A. Hazel, Maria de Fatima Cuembelo, Felix Limbani, Wanderley Bernardo, Fernando Muñoz

**Affiliations:** 1 Centro de Investigación en Sistemas de Salud, National Institute of Public Health, Cuernavaca, México; 2 Independent consultant, Maputo, Mozambique; 3 National Institute of Health, Bogotá, D.C. Colombia; 4 School of Public Health, University of Ghana, Accra, Ghana; 5 Center for Health Policy, School of Public Health, University of the Witwatersrand, Witwatersrand, Republic of South Africa; 6 Dignities International, Research Department, Knowledge Translation Unit, Zomba, Malawi; 7 University of Sao Paolo, Sao Paolo, Brazil; 8 Facultad de Medicina, Universidad de Chile, Santiago, Chile

**Keywords:** Africa, Evidence, Health programs, Inequities, Latin America, Maternal and child health, Research utilization

## Abstract

**Background:**

Striving to foster collaboration among countries suffering from maternal and child health (MCH) inequities, the MASCOT project mapped and analyzed the use of research in strategies tackling them in 11 low- and middle-income countries. This article aims to present the way in which research influenced MCH policies and programs in six of these countries – three in Africa and three in Latin America.

**Methods:**

Qualitative research using a thematic synthesis narrative process was used to identify and describe who is producing what kind of research, how research is funded, how inequities are approached by research and policies, the countries’ research capacities, and the type of evidence base that MCH policies and programs use. Four tools were designed for these purposes: an online survey for researchers, a semi-structured interview with decision makers, and two content analysis guides: one for policy and programs documents and one for scientific articles.

**Results:**

Three modalities of research utilization were observed in the strategies tackling MCH inequities in the six included countries – instrumental, conceptual and symbolic. Instrumental utilization directly relates the formulation and contents of the strategies with research results, and is the least used within the analyzed policies and programs. Even though research is considered as an important input to support decision making and most of the analyzed countries count five or six relevant MCH research initiatives, in most cases, the actual impact of research is not clearly identifiable.

**Conclusions:**

While MCH research is increasing in low- and middle-income countries, the impact of its outcomes on policy formulation is low. We did not identify a direct relationship between the nature of the financial support organizations and the kind of evidence utilization within the policy process. There is still a visible gap between researchers and policymakers regarding their different intentions to link evidence and decision making processes.

## Background

Health inequities continue to be an issue that affects all societies worldwide and significant health status differences can be seen inside as well as between countries, regions, and continents. Inside every country, rich or poor, some inevitable differences in health can be observed across populations, but beyond justifiable inequalities and inequities affecting children, adolescents and mothers remain particularly evident [1, 2].

Health standards, such as infant mortality, maternal mortality or life expectancy, constitute good indicators of these inequities. While neonatal mortality rates were halved in the European and Western Pacific regions between 1990 and 2010, the reduction observed in the African region was only of 19%; progress has been generally slow, mostly in the region with the highest neonatal mortality rates [3]. In 2010, the maternal mortality ratio per 100,000 live births was between 450 and 1500 in Africa, between 62 and 170 in the Americas, and between 17 and 64 in Europe (interagency estimations) [4]. Such large differences are not only explained by geographical and cultural factors; in relation to the prosperity of a society, access to healthcare and the quality of the care people have access to, are but two of many determinants of an unequally distributed health level among populations.

To tackle these inequities, evidence issuing from research activities has been used to support the development and identification of strategies and interventions. Good technical elements and proven effectiveness of certain interventions are not the only needed characteristics for reducing inequities. Furthermore, political will is also necessary to reach redistribution of sources in a country and to assure that interventions become operational [5].

The European Commission funded the Multilateral Association for Studying Health Inequities and Enhancing North-south and South-South Cooperation (MASCOT) programme between 2011 and 2013, which is a multicentre consortium [6]. Besides mapping maternal and child health (MCH) conditions and health research systems capacities, MASCOT also examined the remediation strategies currently aimed at tackling health inequities in eleven low- and middle-income countries (LMICs) from Africa and Latin America to evaluate the use of research evidence for the formulation, planning or reshaping of policies and programs.

Different frameworks have been conceived in order to assess the impact of research in policymaking [7, 8]. Research uptake not only depends on the soundness and pertinence of its results. The particular position of public health researchers among other stakeholders who favour or are against change, particularly the Ministries of Health, is an important factor [9]. Rather than trying to measure the impact of research, this study focused on finding out how research influenced MCH policies and programs. Research results can be used in different ways as support for decision making and, more specifically, in the design, formulation or reshaping of any given policy or program [10]. The MASCOT consortium based its evaluation of strategies tackling MCH inequalities considering three basic ways in which research results had been used in their design, formulation or reshaping, namely instrumental, conceptual and symbolic [11].

The instrumental use of research occurs when research is specially tailored or its results are directly used to answer to a particular health need. In this case, a clear influence of research results can be recognized in the formulation, planning, reshaping or implementation of a given intervention, policy or program. We can distinguish two subcategories of instrumental use of research results: (1) researchers identify a particular need and the uptake of their work is decided by decision makers, and (2) when decision makers or program managers demand the assistance of researchers to look for answers that will support or define the design of a strategy [10, 11]. Conceptual use of research results is made when certain concepts, theories or perspectives developed by research serve to strengthen the formulation of an already made decision [10, 12]. Research exists with no immediate relation to the policy or program in scope, but its results help to sustain them. Finally, symbolic use of research results can be identified in the case of decisions, policies or programs which are made based on arguments that are not necessarily linked to research, but research results are brought up to justify them [11]. In this last case, neither the particular results nor the concepts, theories and ideas of research serve as foundation of the policy or program, but only their ‘scientific aura’ is used to justify them.

The paper aims to present the way in which research influenced MCH policies and programs while describing other topics such as who is producing what kind of research, how research is funded, how inequities are approached by research and policies, the countries’ research capacities, and the type of evidence base that MCH policies and programs use.

## Methods

### Design

**Fig. 1:**
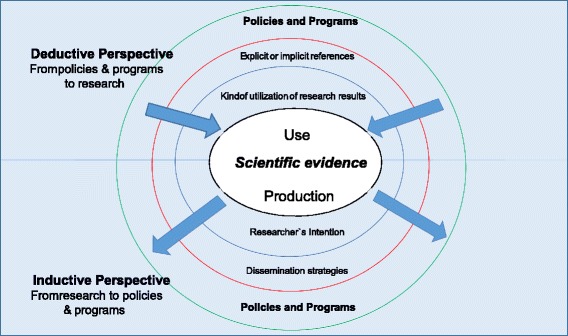
Mental map: Deductive and inductive perspective the use of research. The inductive perspective focused on each country’s scientific production and the manifested direct and indirect intentions of researchers to influence policies. The deductive perspective analyze relevant interventions, always focused in identify the scientific useᅟ

This is a qualitative study using a thematic synthesis narrative process [13]. Four tools using mixed methodologies were developed (Table 1) with two perspectives: inductive and deductive (Fig. 1). The inductive perspective focused on each country’s scientific production and the manifested direct and indirect intentions of researchers to influence policies or in studying or reducing health inequities when formulating or disseminating their work. In this case, we started from research production and tracked the way towards policies and programs (Tools A and D). The deductive perspective analyzed relevant MCH policies and programs that had contributed to the reduction of MCH inequities directly or indirectly and assessed these to track down any research that influenced its design and/or implementation: from policies and programs to research. The main documents related with the design and functioning of these strategies were analyzed and key actors were interviewed to acknowledge if there had been any kind of research uptake to support them (Tools B and C).

**Table 1:** Description of the tools used to identify research production and utilization

Tool	Characteristics	Unit of observation	Variables
A - Intended and unintended project impacts and influencing mechanisms within policy process	Online survey including alternative email responses	Principal investigators	• MCH research organization and production • Equity interventions described • MCH research topic and outcomes • Strategies for diffusion of evidence
B - Evidence use in the formulation of maternal and child health (MCH) policy and programs	Template	MCH policies and program documents	• Policymaking bodies authors • General objective of the MCH policy or program equity addressed • Government unit that has made the decision • Impact on the health system • Type of intervention (single intervention to cross-cutting health system elements) • Research literature refers (implicit references and explicit references) • Ways in which research results had been used within policy process (symbolic, instrumental or conceptual)
	Includes handbook		
C - Perspective of policymakers or program managers about the influence of MCH research production on MCH policy and programs	Semi-structured interview	Policymakers and program managers	• Institution’s role in the MCH policy or program • Main institutions working on and supporting the policy and program formulation and execution • Strength and relevance of MCH research in the policy formulation • Research sources used • When has the use of information based on MCH research been most important (agenda setting, policy formulation, policy implementation or policy assessment) • Type of interventions in equity • Main characteristics of the MCH research that made it useful
	Includes handbook		
D - Strength and relevance nationally and internationally published country-relevant recommendations	Template	Scientific articles published in peer reviewed journals from 2009 to 2012	• Document identification • Journal impact factor • Institutions adscriptions • Type of document • Main subject of the paper • Beneficiaries/issues of the document (demographic group; level of intervention; geographic topic; type of population) • Main thrust or focus of the goals of the document in tackling inequalities
	Includes handbook		

Source: Based on MASCOT internal document Deliverable D4.1 and MASCOT Methodological Guidelines. Available from: (http://cordis.europa.eu/result/rcn/156424_en.html)

The thematic synthesis narrative helped us identify if and how the use of research results is determined by the researchers’ intention to approach a politically relevant issue [13]. We also explored the dissemination strategies used by researchers to influence decision makers. This also made it possible to infer how effective the use of research was in generating policies with an impact on the reduction of MCH inequities.

Finally, the role of research in the process of policies’ formulation was identified through the explicit or implicit use of evidence in relation to quotations and references in the main documents of each policy or program. Once this use was identified, a distinction was drawn regarding the instrumental, conceptual or symbolic use of research by establishing a relationship with the context of decision making and policy or program formulation. Whenever a strategy made direct use of data or information derived from research we coded this kind of use as instrumental. A general use of theories derived from research to support the strategy but with no direct or factual relation was coded as conceptual. Finally, an even more general evocation of scientific production not clearly supporting the strategy was qualified as symbolic use of research.

Each tool was applied and analyzed by the country expert or MASCOT partner and included a handbook with instructions and definitions of inclusion and exclusion criteria for each object of the study and the analysis method for each tool. MASCOT partners and local experts received continuous counselling and technical support. The whole MASCOT project was approved by the research and ethics committees in each institution and country where this was mandatory.

### Setting

The study was carried out from April to December 2012. This paper is focused in six LMICs (three African and three Latin American countries) selected among the 11 countries of the MASCOT project considering the best quality and comparability of their information.

### Coding and analysis

The interviews were transcribed verbatim in EXCEL files and each country expert analysed the transcripts to identify and code themes. The themes were subsequently analysed at a more interpretative level, with constant cross verification with the outcomes from the other tools. Each content analysis tool was consolidated in a WORD file using a synthesis narrative approach. Finally, the results of the online survey were analysed with descriptive statistics.

### Consolidation of country outcomes

Each country expert consolidated their data, analysis, discussion and conclusions into a WORD file. We organized that information in an Excel file to identify common themes and codes. The following codes were identified and registered for each country: country’s official name, policy and/or program analysed, program and policy description, dimensions of the policy and program, research utilization models, MCH research organization and production, equity interventions described in each research paper, MCH research topic and outcomes, strategies for diffusion of evidence, MCH research utilization and impact on policy and program, and main conclusions. The analysis approach was developed using the thematic analysis method [13].

## Results

Several strategies tackling MCH inequities were identified in each one of the six selected countries, three from Africa (Ghana, Malawi and Mozambique) and three from Latin America (Brazil, Chile and Mexico; Tables 2 and 3).

**Table 2:** Main policies and programs tackling maternal and child health (MCH) inequalities, by country 2012^a^

Country	Strategy	Goal	Use of research results
Ghana	Community Health Planning and Services (CHPS)	Improve prevention, treatment and management of diseases to improve MCH, re-orient and relocate primary healthcare to community locations	Instrumental; based on an experiment at the Navrongo Health Research Center in the Kassena-Nankana District
	National Infant and Young Child Feeding (NIYCF) program	Create an environment enabling mothers, families and caregivers to make and implement informed choices about optimal feeding practices for infants and young children	Instrumental; based on the WHO/UNICEF Global Strategy on Infant and Young Child Feeding
	Reproductive Health Service Policy	Develop and distribute appropriate cadres of service providers according to workload, as well geographical and access equity	Symbolic use of evidence
Malawi	The National Sexual and Reproductive Health and Rights Policy 2009 edition	Increasing availability, accessibility, utilization and quality of skilled obstetric care during pregnancy, childbirth and postnatal period	No explicit expression of policy being developed based on research evidence
	The Road Map for Accelerating the Reduction of Maternal and Neonatal Mortality and Morbidity in Malawi 2007–2012	Framework for provision of comprehensive sexual and reproductive health services to the population	Combining the three types of use of research, it followed a National assessment of emergency obstetric care services influenced by the African Union (2004)
Mozambique	National Policy on Health and Sexual and Reproductive Rights	Increase demand for family planning services and contraception; increase commitment and mobilization of resources and strengthen coordination mechanisms	Instrumental and conceptual use of research done for other purposes
	Strategy for the reduction of Maternal and Perinatal Mortality	Increase the use of basic and complete essential obstetric services	Instrumental use of a study on Safe Motherhood Needs Assessment commissioned by WHO
	Strategy for Family Planning and Contraception (2010–2015)	Increase availability and quality of family planning services and contraception; increase demand for family planning services and contraception	Not defined
Brazil	Iniciativa Hospital Amigo da Criança (Child friendly hospital initiative) UNICEF	Implement attention to women’s health and child health with a focus on care during labour, birth, growth and development of children from birth to 24 months; Organizing Network of Care for Maternal and Child Health to assure access, hosting and responsiveness	No explicit use of research results in the formulation of national programs and policies
			The programs and policies use past research as a conceptual support
	Pacto pela Redução da Mortalidade Infantil Nordeste-Amazônia Legal (Infant Mortality Reduction Northeast-Amazon)	Accelerate the reduction of inequalities in the Northeast and in the Amazon, reducing child mortality (children under 1 year of age), especially the neonatal component (up to 27 days old)	
	Política Nacional de Atenção Integral à Saúde da Mulher (National Policy on Comprehensive Health Care for Women)	Reduce morbidity and mortality from cancer in female population; promote the healthcare of black women, the field workers, indigenous women and women in situations of detention, including the promotion of prevention and control of sexually transmitted diseases and HIV/AIDS	
Chile	National Strategic Health Plan	Explicit entitlements for the treatment of prioritized health problems (AUGE), changes in the regulatory scheme of the Health System separating public health activities from health provision, and enforcing the governmental regulation of private and public health insurance and provision of individual health services	Instrumental use was central in the justification and objectives
	Program for Adolescents Care	Improve the demand for adolescent care services and to provide a coherent and integral healthcare	Explicit evidence comes from national experience of the specialists in charge
Mexico	Equal Start in Life (APV)	Strengthens provider capacity and stimulates community participation to support prenatal care and professional delivery	Instrumental role of research in the formulation and implementation of the three programs
	Opportunities	Cash transfers conditioned to children attending school and to mothers and children visiting primary health centres and health promotion interventions aiming to improve MCH and the nutritional status of children	
	Popular Health Insurance (SPS)	Voluntary affiliation health insurance program giving access to a package of medical interventions for families excluded from the social security institutions either in the informal sector of the economy or self-employed	

^a^ Source: Based on MASCOT project Final Reports. Available from: (http://cordis.europa.eu/result/rcn/156424_en.html)

**Table 3:** Main maternal and child health (MCH) research data by country 2012^a^

Country	Top MCH research institutions	Active MCH research projects and production	MCH research funding	MCH research utilization in policies and programs
Ghana	• Ghana Health Service Research and Development Division’s four research centres: NHRC, KHRC, DHRC and OCRC • University of Ghana Medical School • University of Ghana School of Public Health • Noguchi Memorial Institute for Medical Research of the University of Ghana • Department of Obstetrics and Gynecology, Tamale Teaching Hospital	• 216 health active research projects between 2009 and 2011 • 50 on MCH • 40 scientific papers on MCH in PubMed between 2009 and 2012 • 21 on MCH-equity • MASCOT online survey identified 14 articles in peer review journals	Over 90% of research funding from external donors	• MCH research use in the development of MCH policies and programs to reduce inequalities is average, most of the time in instrumental manner
Malawi	• University of Malawi • College of Medicine • University of North Carolina Project • Malaria Alert Centre • Center for Reproductive Health • Dignitas International • Malawi Institute of Management	• 71 health research projects between 2009 and 2012; 33 on MCH	Mostly external funding from international agencies and NGOs	• Limited capacity within the Ministry of Health to gather and use research findings • Research data gathered in Malawi is often analyzed and used in other countries
Mozambique	• No responsible organism in MCH research • Few facilities have access to studies or documented research in MCH	• Very scarce MCH research • No specific data available	Mostly external funding from international agencies and NGOs	• Studies commissioned by WHO have been instrumental in the development of strategies to reduce maternal mortality • Articles resulting from research conducted for other purposes have been used to support the formulation of policies in symbolic manner
Brazil	• IMIP • IPESQ • Epidemiologia UFPEL • Observatório Sobre Iniquidades em Saúde • Ministério da Saúde • Faculdade de Saúde Pública	• 90 references related to research on inequities from 2009 to 2012	Mostly public funding (around 70% in 2007)	• The programs and policies are based on a retroactive use of research; past data and programs are used as support, most of the time in conceptual manner
			Universities and research institutions were the main recipients (55.5%)	
Chile	• Universidad de Chile (UCH) • Catholic University of Chile (PUC) • The University of Santiago • Universidad Austral de Chile • Universidad de Concepción	From 2009 to 2012 identifying 370 references in MCH and inequalities	In 2010 FONDECYT funded about 80% of research	• National and international evidence is used in instrumental way • The principal source of information is the expertise of key actors
			18% FONDES	
			2% FONIS	
Mexico	• National Institute of Public Health • Mexican Institute of Social Security – Distrito Federal • Population Council-Mexico • Mexican Institute of Social Security – Guadalajara • El Colégio de México • National Institute of Pediatrics • Universidad Autónoma de Guerrero	The top ten institutions reported 103 active MCH research projects between 2009 and 2012	Main research funding comes from CONACYT and other national initiatives	• The scientific production between 2009 and 2011 shows a clear tendency to address topics of direct interest for researchers or institutions, it was used in instrumental way

^a^ Source: Based on MASCOT project Final Reports. Available from: (http://www.mascotfp7.eu/mascot-resources/reports/)

Some of them directly address these inequities, while others have more general aims that indirectly affect the health status of mothers and children (Table 2). We present a general description of the kind of MCH research organization and production we found in them, as well as the most relevant policies and programs of each country and the way research is being used in them as the project was able to identify (Table 3).

## Ghana

### MCH research organization and production – inductive perspective

MCH research in Ghana is mainly produced by the Research and Development Division of the Ghana Health Services and its four research centres. Other institutions linked to the University of Ghana and the Tamale Teaching Hospital also account for research efforts; 216 health research projects were active in 2009–2011. Among them, 50 focused on MCH matters. Almost 90% of research funding comes from external donors, mainly the Bill and Melinda Gates Foundation. Other newborn and children projects were funded by WHO, DfID, Save Newborn Lives, Save the Children and the Malaria Ventures Initiative.

The online survey identified at least three research projects likely to involve high impact decision makers and program managers. Researchers on two projects included medium impact decision makers, and eight projects involved other health decision makers and program managers.

A literature search in PubMed identified 40 published scientific papers between 2009 and 2012, among which 21 focused on MCH equity; while the MASCOT online survey identified 14 articles. The nine papers from international peer review journals that were found and analyzed are related to health projects that were active between 2009 and 2012. These papers included seven original research articles, one clinical case report and one short report.

Executive summaries, policy briefs, bulletins and web pages are the commonest dissemination platforms in Ghana. The highest priority targets for diffusion efforts in Ghana are health program managers and health workers.

### Strategies tackling MCH inequities – deductive perspective

The Community-based Health and Planning Services (CHPS), the Reproductive Health Service Policy, the Under Five Child Health Policy (UFCHP), the National Health Insurance Scheme (NHIS) and other policies and programs have been developed to improve MCH in Ghana, but are often challenged by bottlenecks and the main determinants of MCH inequities. The main dimensions of these strategies include equity of access, mobilizing community resource for healthcare, efficiency, disease prevention, quality of care, and health systems management.

The main objectives of the equity-oriented projects on access and healthcare financing were to improve equity in healthcare and provide risk protection to poor households under the NHIS. Some projects also aimed at assessing the feasibility and efficiency of the strategies used in identifying the poor for premium exemptions under the NHIS.

Most MCH policies and programs also draw their evidence from international policy frameworks and other reports from international organizations such as WHO, UNICEF and UNFPA [14, 15]. The National Infant and Young Child Feeding Program is an example developed on 14 explicit references to research results, only four of which were generated in Ghana [14]. While the utilization model for the CHPS policy was mainly instrumental, the use of several follow-up researches has been symbolic and conceptual, aimed at shaping the implementation of the policy at various levels of the health system. Symbolic use of research in policies and programs is significant in Ghana for scaling up health programs.

## Malawi

### MCH research organization and production – inductive perspective

Approximately 70% of the targeted respondents on the online survey had health research projects active between 2009 and 2012, of which almost half were focused on MCH matters. Nevertheless, the project identified limited capacity within the Ministry of Health (MoH) to gather and use up-to-date research findings. On the other hand, research data gathered in Malawi is often analyzed and utilized in other countries to meet their research agendas. There is no national forum for the dissemination of research findings [16].

Among the 10 top research institutions in relation to their scientific papers production, the most relevant are the University of Malawi, the College of Medicine, the University of North Carolina Project, the Malaria Alert Centre, the Center for Reproductive Health Dignities International and the Malawi Institute of Management.

### Strategies tackling MCH inequities – deductive perspective

A third revised version of the Road Map for Accelerating the Reduction of Maternal and Neonatal Mortality and Morbidity in Malawi issued from the African Union call was developed by the MoH in 2007. Among others, specific objectives of the program linked to MCH inequities include increasing the availability, accessibility, utilization and quality of skilled obstetric care during pregnancy, childbirth and the postnatal period at all levels of the healthcare delivery system, and strengthening overall capacities to improve maternal and neonatal health.

The National Sexual and Reproductive Health and Rights Policy aims at providing direction to decision makers and program managers to effectively implement Sexual and Reproductive Health and Rights (SRHR) services, providing guidelines for capacity building for provision of quality SRHR services, and informing and guiding stakeholders and partners on SRHR issues.

These two programs include explicit (cited with clear references) and implicit (vague mentions with no citation) references to research, particularly in their backgrounds and problem definitions. However, program documents and interviews with policymakers made it possible to determine that much of the evidence was from government instituted research studies, documents from UN agencies or other regional bodies and government agencies. Most of the references made in their formulation derived from Malawi Demographic and Health Survey and Multiple Indicator Cluster Survey reports, suggesting a rather symbolic use of research.

## Mozambique

### MCH research organization and production – inductive perspective

In Mozambique, there is neither a research agenda for health nor an institution that would be responsible for research in health. The National Institute of Health has this mandate but is still in process of reorganization to this end. However, most research in the country is done by both public and private institutions as well as NGOs. These institutions have their own agendas but also respond to calls for proposals launched by NGOs, international partners and public ministries including the MoH.

Although research in MCH is low, it has some influence in the formulation of policies because there is a number of indicators and assumptions that are made in politics on the basis of evidence produced by research targeted to specific population groups. Some important issues were listed regarding the access to services and the use and consumption of the services themselves. On the other hand, documentation from WHO and specialized agencies, such as UNFPA and UNICEF, was incorporated into the formulation of policies tackling MCH inequities.

### Strategies tackling MCH inequities – deductive perspective

The Strategies for the Reduction of Maternal and Neonatal Morbidity and Mortality include different elements and were designed to increase the use of basic and complete essential obstetric services for women with obstetric complications in order to assure access to services as well as timely and good quality care. Their basic document was developed after the study on safe Motherhood Needs Assessment, commissioned by WHO to identify the determinants of maternal mortality, was performed. No doubt the results of this study were instrumental in formulating the policy.

The National Policy on Reproductive Health and Rights aims to promote respect and exercise of sexual and reproductive rights among all stakeholders and ensure the provision of evidence-based sexual and reproductive health services at all levels of care. This policy makes a conceptual use of the findings and recommendations found in articles resulting from research conducted for other purposes, but that help to support it.

These policies have had major impacts such as a significant reduction of maternal mortality ratio of 975 per 100,000 live births in 1997 to 408 in 2003. Another result for which information was obtained in interviews with policymakers was the reduction of unsafe abortion, which contributed greatly to the reduction of maternal mortality.

Even though the practice of institutional research is not routine, research to answer certain questions does contribute to the formulation of policies. According to the type of forum where research findings are disseminated, as well as the audience and the type of research, the purpose of dissemination may or may not be to influence policies and programs.

## Brazil

### MCH research organization and production – inductive perspective

Most health researchers (57%) work in universities, while only 6% work in research institutes; the remainder (37%) are connected to the corporate sector. Only a few research institutes dedicate research efforts to studying MCH inequities. The initial MASCOT literature review identified 12 research articles on MCH inequities. These are mainly dedicated to the South and Southeast regions. Nine articles (75%) are original research, while the remaining three are reviews.

Even though there is a significant production of research addressing MCH inequities in Brazil, particularly in certain regions of the country, the formulation of national policies and programs is not making use of it. A barrier or gap seems to exist between these two activities and policies and programs are rather based on past results, be it in relation to the production of data or the experience of already existing programs.

### Strategies tackling MCH inequities – deductive perspective

There are several policies and programs addressing MCH in Brazil. Some focus on MCH promotion or disease prevention, healthcare services management, social participation and empowerment, and human resources for MCH. Some policies deal with jurisdiction and governance or financial and delivery arrangements, while programs such as More Health Rights for All include these two general dimensions. All programs consider the needs of vulnerable populations identifying indicators of vulnerability, and thus also addressing inequities in MCH. The main policymaking body is the MoH except for the program called A Brazil for Children and Teen-agers, which was coordinated by a private non-profit organization (Abring Foundation for Children and Adolescents).

This last program included the participation of healthcare decision-makers, community and researchers, which gave it the strength to cause a positive outcome in minor health conditions associated with a high incidence of infections by *giardia lamblia*. The data generated served as a consistent evidence base for the introduction of rotavirus vaccination as an effective measure to control diarrhoea and help reduce some MCH inequities.

The most important sources of research results used throughout the design processes of policy and programs were evidence from epidemiological data and national experiences with previous programs and policies, data analyses from administrative and institutional health information systems, international projects, and Brazilian and international research programs to promote health. Generally speaking, research was applied for policy formulation (70%), agenda setting (30%), policy implementation (30%), and policy assessment (10%). However, only 50% of the programs searched to close the gap between the needs satisfaction of the poor and vulnerable groups compared with that of those better off.

## Chile

### MCH research organization and production – inductive perspective

According to the National Fund for Science and Technology (FONDECYT) statistics, 80% of its funding is allocated to universities. The University of Chile is the main recipient, followed by the Catholic University of Chile, the University of Santiago, the Universidad Austral de Chile, the Universidad de Concepción, and a couple more universities. Some new private universities recently joined the group of institutions performing serious research in the country.

Research published in indexed journals in the last years is related to subjects in which the MoH has acted based on previous research conducted by the same authors. This is the case of the evaluation of the impact of the addition of folic acid to flour published by a team from the Institute of Nutrition and Food Technology of the University of Chile. In general, MCH research aims dimensions such as access to health services, commitment with international goals, human rights, ethical aspects of interventions addressed to improve MCH, and management of interventions addressed to improve MCH and reduce inequities.

The relatively recent National Fund for Health Research (FONIS) is the only fund explicitly supporting research with possibilities of being used in policy decisions. FONIS has defined a diffusion strategy for those projects considered to be of interest in shaping health policies. The strategy demands the development of activities (meetings, publication, interviews with decision makers) for results diffusion for all funded projects.

Research addressed to support policy decisions is mostly developed responding to the MoH demand, and sometimes is even mandated by the law. This is the case of the studies about costs of the mandatory health plan with explicit entitlements (AUGE) in terms of opportunity, quality and financial protection. Nevertheless, there is an important need of locally produced research pointed to better address health priorities.

### Strategies tackling MCH inequities – deductive perspective

The National Strategic Health Plan was designed to reduce health inequalities, to increase patient satisfaction and to assure the quality of public health interventions. A majority of the interventions in the plan are based on solid scientific evidence, mostly arising from the international literature. However, there are also examples, such as the Program for Adolescents’ Care, fundamentally based on the experience of the specialists in charge.

The kind of research utilization that is found in the National Strategic Plan is mostly instrumental in supporting interventions to reach its objectives. Nevertheless, in its introduction, research is used in a symbolic way, by means of a selection of historic papers.

More recently, the most important evidence considered by health policymakers arises from the Commission on Social Determinants for Health introduced by the World Health Organization. The products and activity of this commission have been closely followed in Chile and Chilean political figures have been part of the group of experts conducting this effort. The new National Strategic Plan for Health includes a cross-sectional objective addressing the reduction of inequities and represents a pathway that clearly needs to be founded in sound research results.

## Mexico

### MCH research organization and production – inductive perspective

The top 10 health research institutions reported 103 MCH research active projects between 2009 and 2012; 30% focus on maternal health, 27% address MCH in the general population, 20% address child health and 30% address MCH in vulnerable populations. The two main themes of research are health systems (21 projects), particularly on human resources and health providers, and social determinants of health (27 projects). We also identified 11 experimental or clinical studies led by the Mexican Social Security Institute.

Most MCH research is not directly focused on tackling health inequalities, but mainly oriented towards the improvement of the health conditions of the poor, and not towards strategies to reduce gaps between populations with different socioeconomic status.

The main research diffusion mechanisms are researcher driven, basically scientific journals and conferences, with very scant efforts in broader dissemination mechanisms such as executive summaries, newsletters or blogs.

### Strategies tackling MCH inequities – deductive perspective

The most salient program focusing on MCH is Equal Start in Life (APV), launched in 2002. APV strengthens provider capacity and stimulates community participation to support prenatal care and professional delivery. Another important program, known as Oportunidades (presently PROSPERA) is a conditional cash transfer scheme for poor households aiming to interrupt inter-generational transmission of poverty. Along with schooling conditions, Oportunidades conditions the cash transfers to visits by mothers and children to primary health centres and health promotion interventions to improve MCH and the nutritional status of children. A third national program is the Popular Health Insurance (SPS), a voluntary affiliation program to access a package of medical interventions open to families excluded from the social security institutions.

These three programs were formulated using evidence based methodologies. However, their decision making contexts differed significantly: APV was formulated and implemented within the MoH and was subjected mostly to technical appraisals within the bounds of existing budgets. Oportunidades was formulated within the Ministry for Social Development, and required important consultations and co-ordination with the MoH. The SPS implied a change to the General Health Law and required a congressional debate. Among the explicit references to research in the documents of these policies, 13 out of 20 aim to reach equity by promoting the reduction of health gaps by improving insurance, coverage conditions and financial healthcare protection for the poor.

The analysis suggests a mostly conceptual but also somewhat instrumental role of research in the formulation and implementation of the three programs. All three have made instrumental use of research evaluations to guide and adapt implementation and to validate and support policies, particularly when the outgoing federal administration prepared the programs to withstand appraisal by new and possibly not too favourably inclined authorities.

## Discussion

This paper tried to determine how much and in what manner policies and programs tackling MCH inequities are using research in three African and three Latin American LMICs. We found an increasing interest in supporting these strategies with the use of research results. The ways in which these results are used varies from a common, merely symbolic reference to their scientific aura, to the actual use of data specifically produced to inform and orient their design and implementation and passing through the use of their general concepts to give a theoretical support for the strategies.

Generally speaking, the main obstacles hindering the instrumental use of research in the formulation of health policies and programs are the reduced number of researchers working on priority health problems and poor researcher–policymaker communication [17, 18]. The limiting factors are linked to the lack of resources or the researchers’ scientific or professional interests. Poor researcher–policymaker communication also stems from the difference in agendas separately rooted in academia or politics [19–21]. In most cases, the research financers’ objectives prevail, leaving aside particular national needs, except in cases where national needs coincide with research financers’ objectives [22, 23].

In other cases, the decision makers’ choice, which is restricted within an electoral cycle and motivated by re-election, may supersede the long-term health objectives of the researcher [24, 25]. In the case of the African countries of this study we found an important heterogeneity. While in Malawi and Ghana there is an important relationship between research and the countries’ needs, in Mozambique MCH research production is so small that it can barely influence policies.

In the case of the countries where research is linked to MCH and inequities needs, we find diffusion strategies of its results as well as interesting methods to present them to decision makers to impact policy formulation. These methods are adopted in the hopes of presenting research results in a simple and efficient manner that will reduce the time policymakers require to access and assimilate research results.

The three Latin American countries analyzed in this paper account for the largest scientific production in the sub-continent in health research, particularly MCH research. They have experienced research institutions and their government agencies provide guidance and financing for research. Nevertheless, a gap still exists between the generation of knowledge and a more agile and consistent utilization of its results in the design and implementation of strategies tackling MCH inequities.

The process of health research utilization depends on the activities of a wide range of actors, including health professionals, researchers, the public, policymakers and research funders. These actors’ abilities to create a pull for research findings, to engage in linkage and exchange between agencies, researchers and decision makers, or to push results to various audiences differed from one country to the other [26, 27]. In general, a better uptake of research results depends on balancing the competing pressures faced by a national health research system, among which some of the most relevant are basic versus applied research; public versus private research; health needs versus political interests; national versus international funding; public versus private funding, etc. In the best scenario, these criteria should also respond to the health needs in each country [28]. A wider discussion on how MCH research is financed is presented by Footman et al. [29].

## Conclusions

The need to use research results to support decision making is recognized in all the countries of the study and research production is increasing in these LMICs. Nevertheless, the impact of their outcomes within policy formulation is exceptionally low – some countries in Africa have a conceptual use of evidence performed for multilateral organizations which goals are not related with the policy issues, while in Latin America, there are better experiences in instrumental and conceptual models used in the policy formulation.

We did not identify a direct relation between the nature of the organizations financing research and the kind of evidence utilization by the policies and programs. Nevertheless, a more consistent use of research is assured when it is funded by the governments or multilateral organizations.

We reaffirm that different factors, first of all the lack of resources and of a research oriented culture among decision makers, hinder the real instrumental use of research results. Researchers lack incentives and an established culture that could strengthen their direct and indirect intentions to influence policies and to study MCH inequities. This explains why research impact on policy formulation is still small and not easy to track.

## Limitations

While the article opens a way to look at the existence of the essential link between MCH research production and its uptake, an important limitation comes from the small selection of countries, which might bias its findings.

## Recommendations

A closer contact of researchers with decision makers can enhance the latter’s awareness of how research results can better inform and support the design and implementation of policies and programs. This can be fostered by renewed efforts of research to deepen the understanding of how its uptake is happening.

Finally, considering the importance of funding, the participation of international agencies and a more locally oriented agenda of research financing are needed to foster production, demand and an effective utilization of research in the design and implementation of strategies tackling MCH inequities as is the case in all other kinds of health programs.
