# Erratum to: Mapping the use of research to support strategies tackling maternal and child health inequities: evidence from six countries in Africa and Latin America

**DOI:** 10.1186/s12961-016-0099-y

**Published:** 2016-04-06

**Authors:** Emily M. Vargas-Riaño, Victor Becerril-Montekio, Miguel Ángel Gonzalez-Block, Patricia Akweongo, Cynthia N. A. Hazel, Maria de Fatima Cuembelo, Felix Limbani, Wanderley Bernardo, Fernando Muñoz

**Affiliations:** Centro de Investigación en Sistemas de Salud, National Institute of Public Health, Cuernavaca, México; National Institute of Health, Bogotá, D.C. Colombia; School of Public Health, University of Ghana, Cuernavaca, México; Independent consultant, Maputo, Mozambique; Center for Health Policy, School of Public Health, University of the Witwatersrand, Witwatersrand, Republic of South Africa; Dignities International, Research Department, Knowledge Translation Unit, Zomba, Malawi; University of Sao Paolo, Sao Paolo, Brazil; Facultad de Medicina, Universidad de Chile, Santiago, Chile

Unfortunately, the original version of this article [1] contained an error. One of the author’s full names was not included properly. It is included here correctly. The name of the author is Emily M. Vargas-Riaño.
